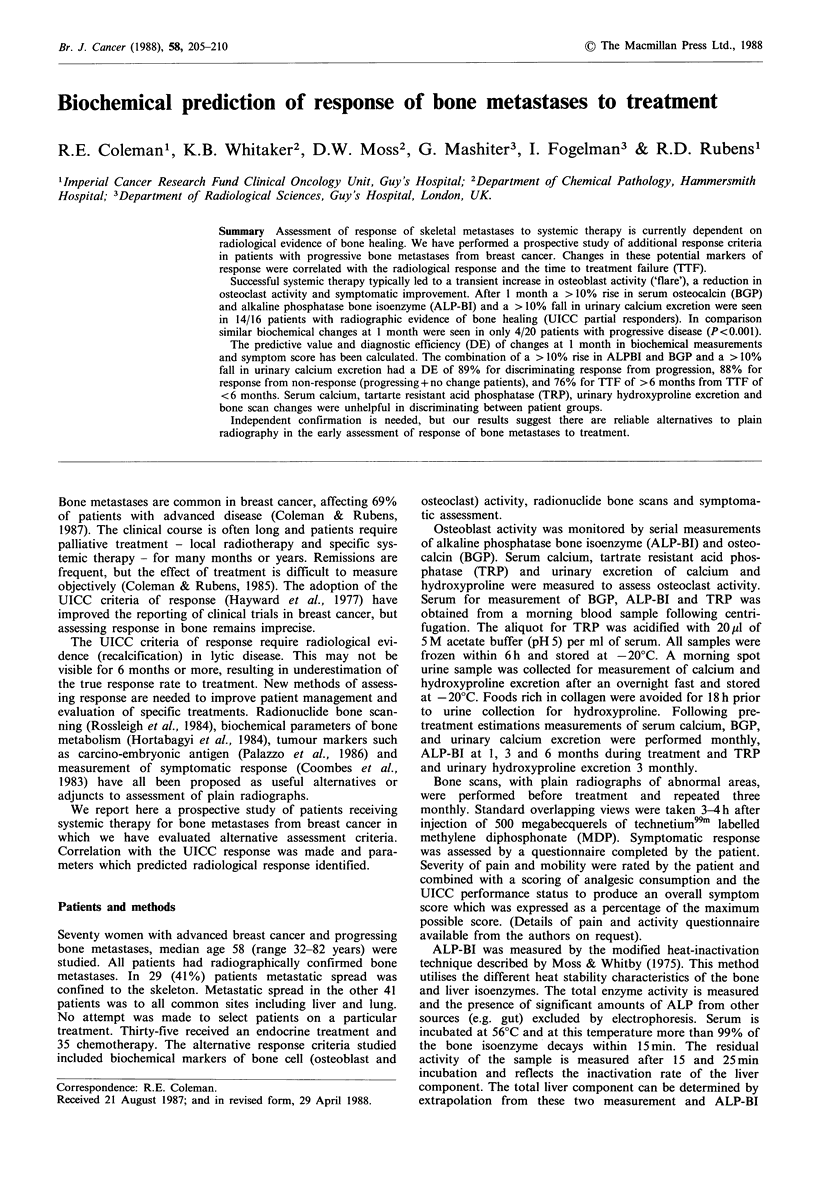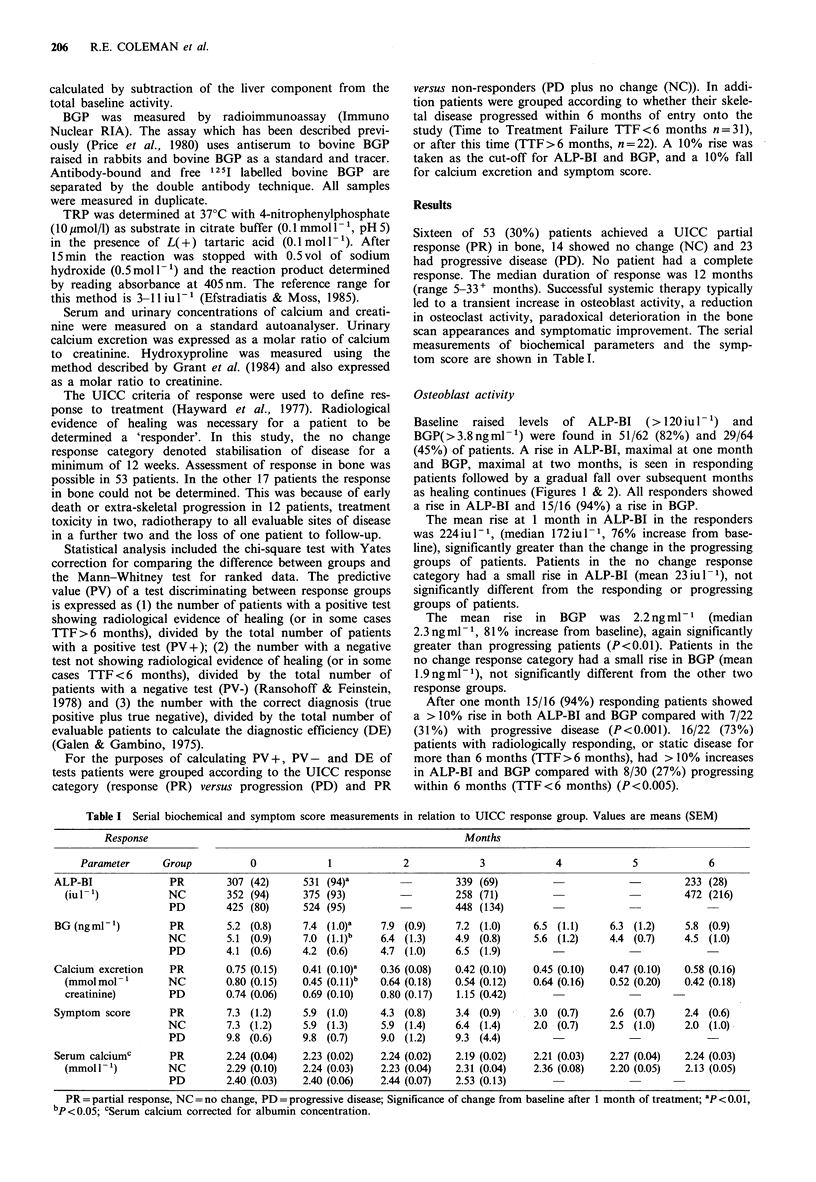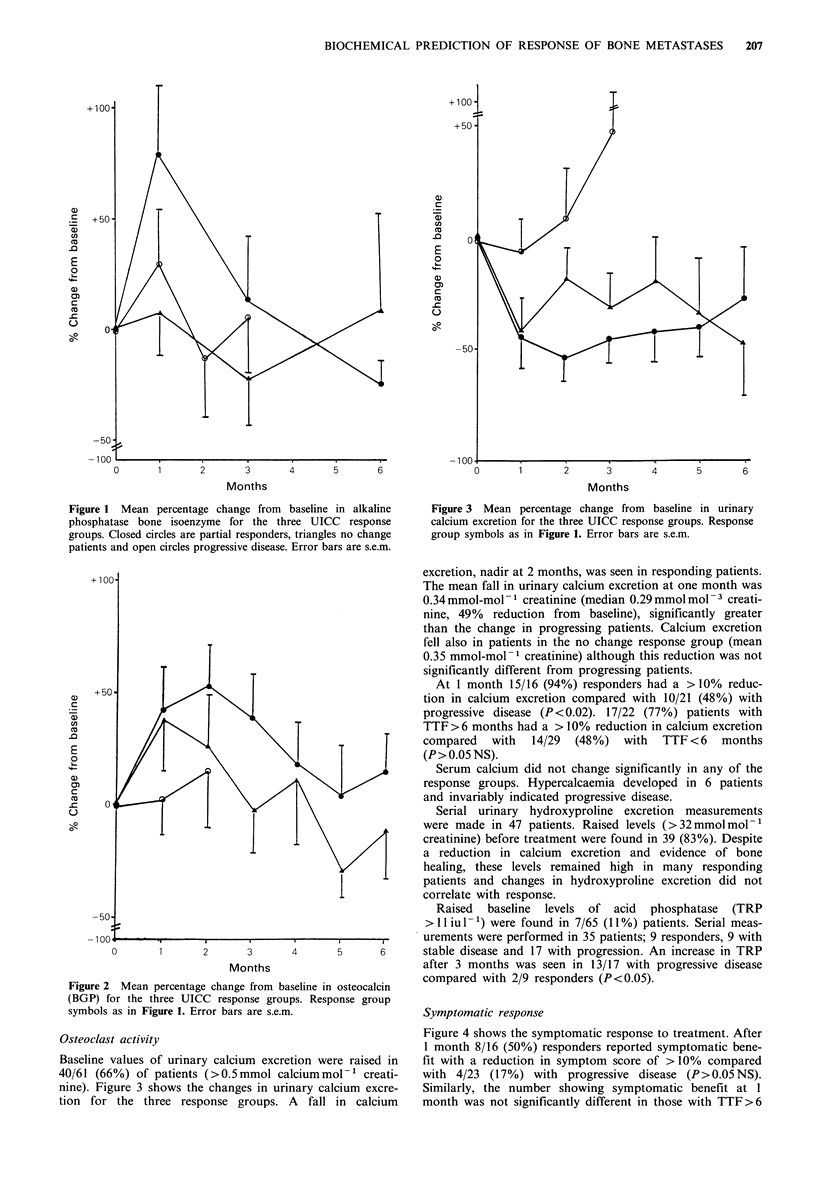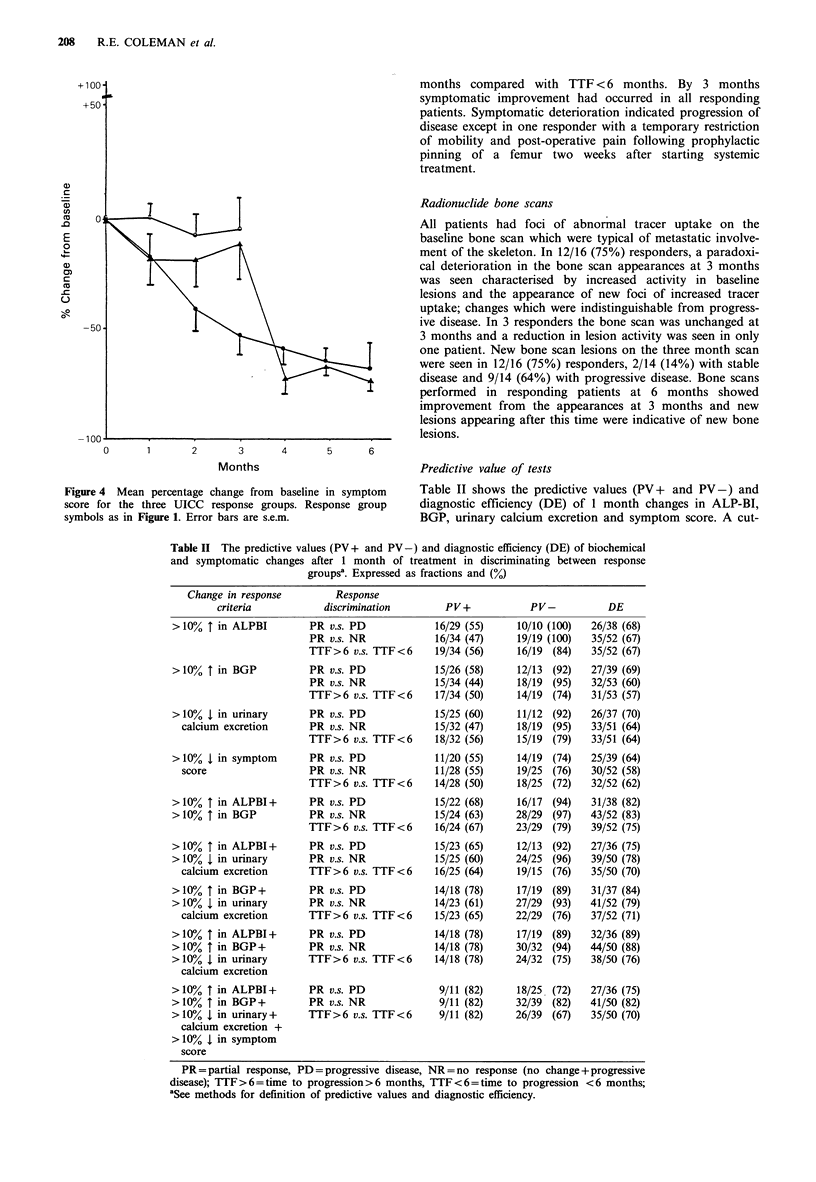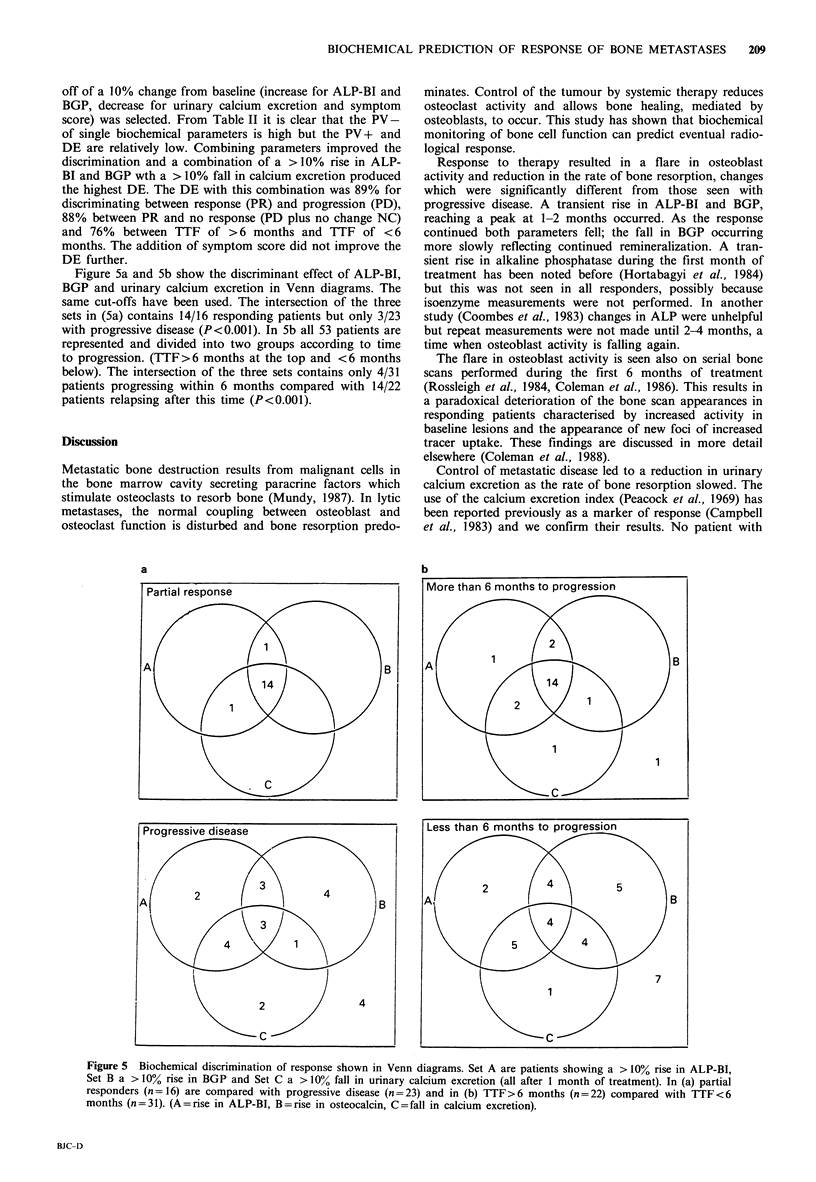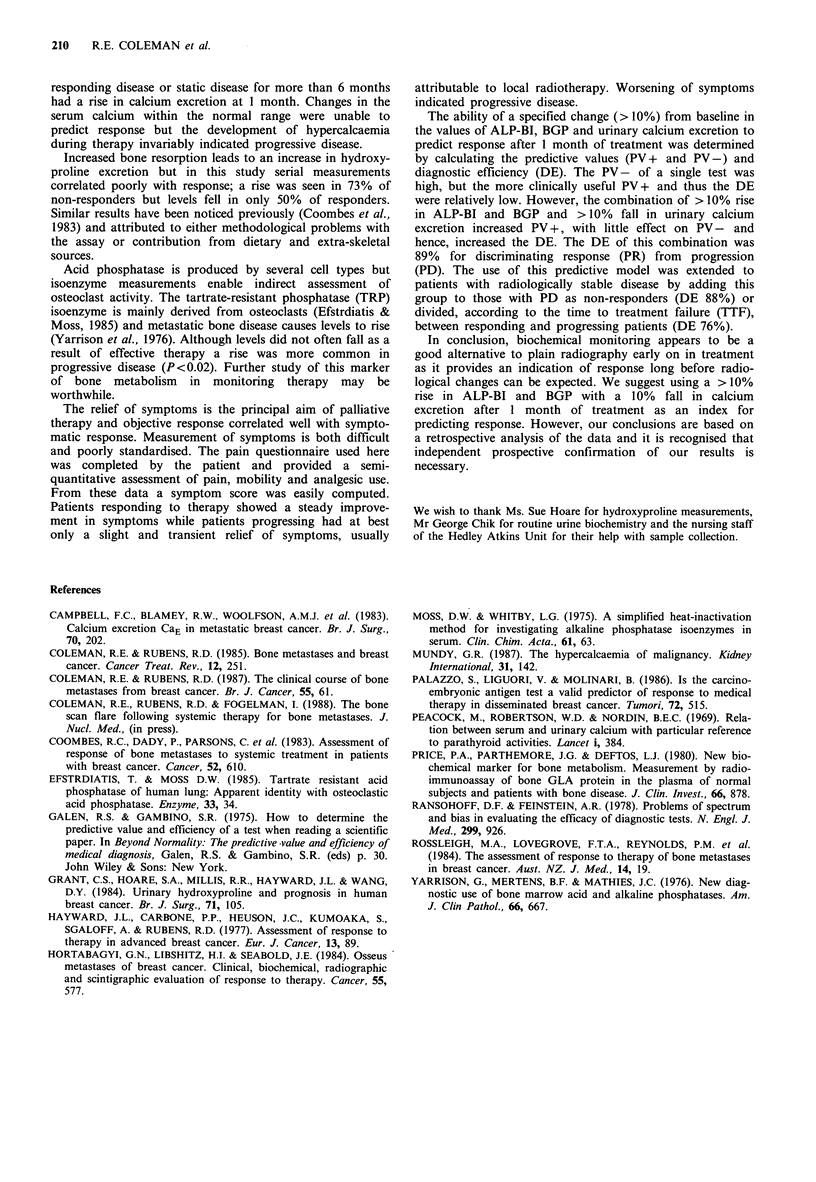# Biochemical prediction of response of bone metastases to treatment.

**DOI:** 10.1038/bjc.1988.194

**Published:** 1988-08

**Authors:** R. E. Coleman, K. B. Whitaker, D. W. Moss, G. Mashiter, I. Fogelman, R. D. Rubens

**Affiliations:** Imperial Cancer Research Fund Clinical Oncology Unit, Guy's Hospital, London, UK.

## Abstract

Assessment of response of skeletal metastases to systemic therapy is currently dependent on radiological evidence of bone healing. We have performed a prospective study of additional response criteria in patients with progressive bone metastases from breast cancer. Changes in these potential markers of response were correlated with the radiological response and the time to treatment failure (TTF). Successful systemic therapy typically led to a transient increase in osteoblast activity ('flare'), a reduction in osteoclast activity and symptomatic improvement. After 1 month a greater than 10% rise in serum osteocalcin (BGP) and alkaline phosphatase bone isoenzyme (ALP-BI) and a greater than 10% fall in urinary calcium excretion were seen in 14/16 patients with radiographic evidence of bone healing (UICC partial responders). In comparison similar biochemical changes at 1 month were seen in only 4/20 patients with progressive disease (P less than 0.001). The predictive value and diagnostic efficiency (DE) of changes at 1 month in biochemical measurements and symptom score has been calculated. The combination of a greater than 10% rise in ALPBI and BGP and a greater than 10% fall in urinary calcium excretion had a DE of 89% for discriminating response from progression, 88% for response from non-response (progressing + no change patients), and 76% for TTF of greater than 6 months from TTF of less than 6 months. Serum calcium, tartrate resistant acid phosphatase (TRP), urinary hydroxyproline excretion and bone scan changes were unhelpful in discriminating between patient groups. Independent confirmation is needed, but our results suggest there are reliable alternatives to plain radiography in the early assessment of response of bone metastases to treatment.


Br. J. Cancer (1988), 58, 205–210 © The Macmillan Press Ltd., 1988

### Biochemical prediction of response of bone metastases to treatment

R.E. Coleman[1], K.B. Whitaker[2], D.W. Moss[2], G. Mashiter[3], I. Fogelman[3] & R.D. Rubens[1]

[1]*Imperial Cancer Research Fund Clinical Oncology Unit, Guy's Hospital;* [2]*Department of Chemical Pathology, Hammersmith Hospital;* [3]*Department of Radiological Sciences, Guy's Hospital, London, UK.*

**Summary** Assessment of response of skeletal metastases to systemic therapy is currently dependent on radiological evidence of bone healing. We have performed a prospective study of additional response criteria in patients with progressive bone metastases from breast cancer. Changes in these potential markers of response were correlated with the radiological response and the time to treatment failure (TTF).

Successful systemic therapy typically led to a transient increase in osteoblast activity ('flare'), a reduction in osteoclast activity and symptomatic improvement. After 1 month a >10% rise in serum osteocalcin (BGP) and alkaline phosphatase bone isoenzyme (ALP-BI) and a >10% fall in urinary calcium excretion were seen in 14/16 patients with radiographic evidence of bone healing (UICC partial responders). In comparison similar biochemical changes at 1 month were seen in only 4/20 patients with progressive disease ($P<0.001$).

The predictive value and diagnostic efficiency (DE) of changes at 1 month in biochemical measurements and symptom score has been calculated. The combination of a >10% rise in ALPBI and BGP and a >10% fall in urinary calcium excretion had a DE of 89% for discriminating response from progression, 88% for response from non-response (progressing + no change patients), and 76% for TTF of >6 months from TTF of <6 months. Serum calcium, tartarte resistant acid phosphatase (TRP), urinary hydroxyproline excretion and bone scan changes were unhelpful in discriminating between patient groups.

Independent confirmation is needed, but our results suggest there are reliable alternatives to plain radiography in the early assessment of response of bone metastases to treatment.

Bone metastases are common in breast cancer, affecting 69% of patients with advanced disease (Coleman & Rubens, 1987). The clinical course is often long and patients require palliative treatment – local radiotherapy and specific systemic therapy – for many months or years. Remissions are frequent, but the effect of treatment is difficult to measure objectively (Coleman & Rubens, 1985). The adoption of the UICC criteria of response (Hayward *et al.*, 1977) have improved the reporting of clinical trials in breast cancer, but assessing response in bone remains imprecise.

The UICC criteria of response require radiological evidence (recalcification) in lytic disease. This may not be visible for 6 months or more, resulting in underestimation of the true response rate to treatment. New methods of assessing response are needed to improve patient management and evaluation of specific treatments. Radionuclide bone scanning (Rossleigh *et al.*, 1984), biochemical parameters of bone metabolism (Hortabagyi *et al.*, 1984), tumour markers such as carcino-embryonic antigen (Palazzo *et al.*, 1986) and measurement of symptomatic response (Coombes *et al.*, 1983) have all been proposed as useful alternatives or adjuncts to assessment of plain radiographs.

We report here a prospective study of patients receiving systemic therapy for bone metastases from breast cancer in which we have evaluated alternative assessment criteria. Correlation with the UICC response was made and parameters which predicted radiological response identified.

### Patients and methods

Seventy women with advanced breast cancer and progressing bone metastases, median age 58 (range 32–82 years) were studied. All patients had radiographically confirmed bone metastases. In 29 (41%) patients metastatic spread was confined to the skeleton. Metastatic spread in the other 41 patients was to all common sites including liver and lung. No attempt was made to select patients on a particular treatment. Thirty-five received an endocrine treatment and 35 chemotherapy. The alternative response criteria studied included biochemical markers of bone cell (osteoblast and osteoclast) activity, radionuclide bone scans and symptomatic assessment.

Osteoblast activity was monitored by serial measurements of alkaline phosphatase bone isoenzyme (ALP-BI) and osteocalcin (BGP). Serum calcium, tartrate resistant acid phosphatase (TRP) and urinary excretion of calcium and hydroxyproline were measured to assess osteoclast activity. Serum for measurement of BGP, ALP-BI and TRP was obtained from a morning blood sample following centrifugation. The aliquot for TRP was acidified with 20 $\mu$l of 5 M acetate buffer (pH 5) per ml of serum. All samples were frozen within 6 h and stored at −20°C. A morning spot urine sample was collected for measurement of calcium and hydroxyproline excretion after an overnight fast and stored at −20°C. Foods rich in collagen were avoided for 18 h prior to urine collection for hydroxyproline. Following pretreatment estimations measurements of serum calcium, BGP, and urinary calcium excretion were performed monthly, ALP-BI at 1, 3 and 6 months during treatment and TRP and urinary hydroxyproline excretion 3 monthly.

Bone scans, with plain radiographs of abnormal areas, were performed before treatment and repeated three monthly. Standard overlapping views were taken 3–4 h after injection of 500 megabecquerels of technetium$^{99m}$ labelled methylene diphosphonate (MDP). Symptomatic response was assessed by a questionnaire completed by the patient. Severity of pain and mobility were rated by the patient and combined with a scoring of analgesic consumption and the UICC performance status to produce an overall symptom score which was expressed as a percentage of the maximum possible score. (Details of pain and activity questionnaire available from the authors on request).

ALP-BI was measured by the modified heat-inactivation technique described by Moss & Whitby (1975). This method utilises the different heat stability characteristics of the bone and liver isoenzymes. The total enzyme activity is measured and the presence of significant amounts of ALP from other sources (e.g. gut) excluded by electrophoresis. Serum is incubated at 56°C and at this temperature more than 99% of the bone isoenzyme decays within 15 min. The residual activity of the sample is measured after 15 and 25 min incubation and reflects the inactivation rate of the liver component. The total liver component can be determined by extrapolation from these two measurement and ALP-BI

Correspondence: R.E. Coleman.
Received 21 August 1987; and in revised form, 29 April 1988.

calculated by subtraction of the liver component from the total baseline activity.

BGP was measured by radioimmunoassay (Immuno Nuclear RIA). The assay which has been described previously (Price et al., 1980) uses antiserum to bovine BGP raised in rabbits and bovine BGP as a standard and tracer. Antibody-bound and free $^{125}$I labelled bovine BGP are separated by the double antibody technique. All samples were measured in duplicate.

TRP was determined at 37°C with 4-nitrophenylphosphate (10 $\mu$mol/l) as substrate in citrate buffer (0.1 mmol l$^{-1}$, pH 5) in the presence of $L(+)$ tartaric acid (0.1 mol l$^{-1}$). After 15 min the reaction was stopped with 0.5 vol of sodium hydroxide (0.5 mol l$^{-1}$) and the reaction product determined by reading absorbance at 405 nm. The reference range for this method is 3–11 iu l$^{-1}$ (Efstradiatis & Moss, 1985).

Serum and urinary concentrations of calcium and creatinine were measured on a standard autoanalyser. Urinary calcium excretion was expressed as a molar ratio of calcium to creatinine. Hydroxyproline was measured using the method described by Grant et al. (1984) and also expressed as a molar ratio to creatinine.

The UICC criteria of response were used to define response to treatment (Hayward et al., 1977). Radiological evidence of healing was necessary for a patient to be determined a 'responder'. In this study, the no change response category denoted stabilisation of disease for a minimum of 12 weeks. Assessment of response in bone was possible in 53 patients. In the other 17 patients the response in bone could not be determined. This was because of early death or extra-skeletal progression in 12 patients, treatment toxicity in two, radiotherapy to all evaluable sites of disease in a further two and the loss of one patient to follow-up.

Statistical analysis included the chi-square test with Yates correction for comparing the difference between groups and the Mann–Whitney test for ranked data. The predictive value (PV) of a test discriminating between response groups is expressed as (1) the number of patients with a positive test showing radiological evidence of healing (or in some cases TTF > 6 months), divided by the total number of patients with a positive test (PV+); (2) the number with a negative test not showing radiological evidence of healing (or in some cases TTF < 6 months), divided by the total number of patients with a negative test (PV-) (Ransohoff & Feinstein, 1978) and (3) the number with the correct diagnosis (true positive plus true negative), divided by the total number of evaluable patients to calculate the diagnostic efficiency (DE) (Galen & Gambino, 1975).

For the purposes of calculating PV+, PV− and DE of tests patients were grouped according to the UICC response category (response (PR) *versus* progression (PD) and PR *versus* non-responders (PD plus no change (NC)). In addition patients were grouped according to whether their skeletal disease progressed within 6 months of entry onto the study (Time to Treatment Failure TTF < 6 months $n=31$), or after this time (TTF > 6 months, $n=22$). A 10% rise was taken as the cut-off for ALP-BI and BGP, and a 10% fall for calcium excretion and symptom score.

### Results

Sixteen of 53 (30%) patients achieved a UICC partial response (PR) in bone, 14 showed no change (NC) and 23 had progressive disease (PD). No patient had a complete response. The median duration of response was 12 months (range 5–33$^{+}$ months). Successful systemic therapy typically led to a transient increase in osteoblast activity, a reduction in osteoclast activity, paradoxical deterioration in the bone scan appearances and symptomatic improvement. The serial measurements of biochemical parameters and the symptom score are shown in Table I.

### *Osteoblast activity*

Baseline raised levels of ALP-BI (> 120 iu l$^{-1}$) and BGP(> 3.8 ng ml$^{-1}$) were found in 51/62 (82%) and 29/64 (45%) of patients. A rise in ALP-BI, maximal at one month and BGP, maximal at two months, is seen in responding patients followed by a gradual fall over subsequent months as healing continues (Figures 1 & 2). All responders showed a rise in ALP-BI and 15/16 (94%) a rise in BGP.

The mean rise at 1 month in ALP-BI in the responders was 224 iu l$^{-1}$, (median 172 iu l$^{-1}$, 76% increase from baseline), significantly greater than the change in the progressing groups of patients. Patients in the no change response category had a small rise in ALP-BI (mean 23 iu l$^{-1}$), not significantly different from the responding or progressing groups of patients.

The mean rise in BGP was 2.2 ng ml$^{-1}$ (median 2.3 ng ml$^{-1}$, 81% increase from baseline), again significantly greater than progressing patients ($P<0.01$). Patients in the no change response category had a small rise in BGP (mean 1.9 ng ml$^{-1}$), not significantly different from the other two response groups.

After one month 15/16 (94%) responding patients showed a > 10% rise in both ALP-BI and BGP compared with 7/22 (31%) with progressive disease ($P<0.001$). 16/22 (73%) patients with radiologically responding, or static disease for more than 6 months (TTF > 6 months), had > 10% increases in ALP-BI and BGP compared with 8/30 (27%) progressing within 6 months (TTF < 6 months) ($P<0.005$).

**Table I** Serial biochemical and symptom score measurements in relation to UICC response group. Values are means (SEM)

| *Response* | | *Months* | | | | | | |
|---|---|---|---|---|---|---|---|---|
| *Parameter* | *Group* | 0 | 1 | 2 | 3 | 4 | 5 | 6 |
| ALP-BI | PR | 307 (42) | 531 (94)[a] | — | 339 (69) | — | — | 233 (28) |
| (iu l$^{-1}$) | NC | 352 (94) | 375 (93) | — | 258 (71) | — | — | 472 (216) |
| | PD | 425 (80) | 524 (95) | — | 448 (134) | — | — | — |
| BG (ng ml$^{-1}$) | PR | 5.2 (0.8) | 7.4 (1.0)[a] | 7.9 (0.9) | 7.2 (1.0) | 6.5 (1.1) | 6.3 (1.2) | 5.8 (0.9) |
| | NC | 5.1 (0.9) | 7.0 (1.1)[b] | 6.4 (1.3) | 4.9 (0.8) | 5.6 (1.2) | 4.4 (0.7) | 4.5 (1.0) |
| | PD | 4.1 (0.6) | 4.2 (0.6) | 4.7 (1.0) | 6.5 (1.9) | — | — | — |
| Calcium excretion | PR | 0.75 (0.15) | 0.41 (0.10)[a] | 0.36 (0.08) | 0.42 (0.10) | 0.45 (0.10) | 0.47 (0.10) | 0.58 (0.16) |
| (mmol mol$^{-1}$ | NC | 0.80 (0.15) | 0.45 (0.11)[b] | 0.64 (0.18) | 0.54 (0.12) | 0.64 (0.16) | 0.52 (0.20) | 0.42 (0.18) |
| creatinine) | PD | 0.74 (0.06) | 0.69 (0.10) | 0.80 (0.17) | 1.15 (0.42) | — | — | — |
| Symptom score | PR | 7.3 (1.2) | 5.9 (1.0) | 4.3 (0.8) | 3.4 (0.9) | 3.0 (0.7) | 2.6 (0.7) | 2.4 (0.6) |
| | NC | 7.3 (1.2) | 5.9 (1.3) | 5.9 (1.4) | 6.4 (1.4) | 2.0 (0.7) | 2.5 (1.0) | 2.0 (1.0) |
| | PD | 9.8 (0.6) | 9.8 (0.7) | 9.0 (1.2) | 9.3 (4.4) | — | — | — |
| Serum calcium[c] | PR | 2.24 (0.04) | 2.23 (0.02) | 2.24 (0.02) | 2.19 (0.02) | 2.21 (0.03) | 2.27 (0.04) | 2.24 (0.03) |
| (mmol l$^{-1}$) | NC | 2.29 (0.10) | 2.24 (0.03) | 2.23 (0.04) | 2.31 (0.04) | 2.36 (0.08) | 2.20 (0.05) | 2.13 (0.05) |
| | PD | 2.40 (0.03) | 2.40 (0.06) | 2.44 (0.07) | 2.53 (0.13) | — | — | — |

PR = partial response, NC = no change, PD = progressive disease; Significance of change from baseline after 1 month of treatment; [a]$P<0.01$, [b]$P<0.05$; [c]Serum calcium corrected for albumin concentration.

**Figure 1** Mean percentage change from baseline in alkaline phosphatase bone isoenzyme for the three UICC response groups. Closed circles are partial responders, triangles no change patients and open circles progressive disease. Error bars are s.e.m.

**Figure 2** Mean percentage change from baseline in osteocalcin (BGP) for the three UICC response groups. Response group symbols as in **Figure 1.** Error bars are s.e.m.

*Osteoclast activity*

Baseline values of urinary calcium excretion were raised in 40/61 (66%) of patients (>0.5 mmol calcium mol$^{-1}$ creatinine). Figure 3 shows the changes in urinary calcium excretion for the three response groups. A fall in calcium

**Figure 3** Mean percentage change from baseline in urinary calcium excretion for the three UICC response groups. Response group symbols as in **Figure 1.** Error bars are s.e.m.

excretion, nadir at 2 months, was seen in responding patients. The mean fall in urinary calcium excretion at one month was 0.34 mmol-mol$^{-1}$ creatinine (median 0.29 mmol mol$^{-3}$ creatinine, 49% reduction from baseline), significantly greater than the change in progressing patients. Calcium excretion fell also in patients in the no change response group (mean 0.35 mmol-mol$^{-1}$ creatinine) although this reduction was not significantly different from progressing patients.

At 1 month 15/16 (94%) responders had a >10% reduction in calcium excretion compared with 10/21 (48%) with progressive disease ($P$<0.02). 17/22 (77%) patients with TTF>6 months had a >10% reduction in calcium excretion compared with 14/29 (48%) with TTF<6 months ($P$>0.05 NS).

Serum calcium did not change significantly in any of the response groups. Hypercalcaemia developed in 6 patients and invariably indicated progressive disease.

Serial urinary hydroxyproline excretion measurements were made in 47 patients. Raised levels (>32 mmol mol$^{-1}$ creatinine) before treatment were found in 39 (83%). Despite a reduction in calcium excretion and evidence of bone healing, these levels remained high in many responding patients and changes in hydroxyproline excretion did not correlate with response.

Raised baseline levels of acid phosphatase (TRP >11 iu l$^{-1}$) were found in 7/65 (11%) patients. Serial measurements were performed in 35 patients; 9 responders, 9 with stable disease and 17 with progression. An increase in TRP after 3 months was seen in 13/17 with progressive disease compared with 2/9 responders ($P$<0.05).

*Symptomatic response*

Figure 4 shows the symptomatic response to treatment. After 1 month 8/16 (50%) responders reported symptomatic benefit with a reduction in symptom score of >10% compared with 4/23 (17%) with progressive disease ($P$>0.05 NS). Similarly, the number showing symptomatic benefit at 1 month was not significantly different in those with TTF>6

**Figure 4** Mean percentage change from baseline in symptom score for the three UICC response groups. Response group symbols as in **Figure 1**. Error bars are s.e.m.

months compared with TTF<6 months. By 3 months symptomatic improvement had occurred in all responding patients. Symptomatic deterioration indicated progression of disease except in one responder with a temporary restriction of mobility and post-operative pain following prophylactic pinning of a femur two weeks after starting systemic treatment.

*Radionuclide bone scans*

All patients had foci of abnormal tracer uptake on the baseline bone scan which were typical of metastatic involvement of the skeleton. In 12/16 (75%) responders, a paradoxical deterioration in the bone scan appearances at 3 months was seen characterised by increased activity in baseline lesions and the appearance of new foci of increased tracer uptake; changes which were indistinguishable from progressive disease. In 3 responders the bone scan was unchanged at 3 months and a reduction in lesion activity was seen in only one patient. New bone scan lesions on the three month scan were seen in 12/16 (75%) responders, 2/14 (14%) with stable disease and 9/14 (64%) with progressive disease. Bone scans performed in responding patients at 6 months showed improvement from the appearances at 3 months and new lesions appearing after this time were indicative of new bone lesions.

*Predictive value of tests*

Table II shows the predictive values (PV+ and PV−) and diagnostic efficiency (DE) of 1 month changes in ALP-BI, BGP, urinary calcium excretion and symptom score. A cut-

**Table II** The predictive values (PV+ and PV−) and diagnostic efficiency (DE) of biochemical and symptomatic changes after 1 month of treatment in discriminating between response groups[a]. Expressed as fractions and (%)

| *Change in response criteria* | *Response discrimination* | *PV+* | *PV−* | *DE* |
|---|---|---|---|---|
| >10% ↑ in ALPBI | PR *v.s.* PD | 16/29 (55) | 10/10 (100) | 26/38 (68) |
| | PR *v.s.* NR | 16/34 (47) | 19/19 (100) | 35/52 (67) |
| | TTF>6 *v.s.* TTF<6 | 19/34 (56) | 16/19 (84) | 35/52 (67) |
| >10% ↑ in BGP | PR *v.s.* PD | 15/26 (58) | 12/13 (92) | 27/39 (69) |
| | PR *v.s.* NR | 15/34 (44) | 18/19 (95) | 32/53 (60) |
| | TTF>6 *v.s.* TTF<6 | 17/34 (50) | 14/19 (74) | 31/53 (57) |
| >10% ↓ in urinary calcium excretion | PR *v.s.* PD | 15/25 (60) | 11/12 (92) | 26/37 (70) |
| | PR *v.s.* NR | 15/32 (47) | 18/19 (95) | 33/51 (64) |
| | TTF>6 *v.s.* TTF<6 | 18/32 (56) | 15/19 (79) | 33/51 (64) |
| >10% ↓ in symptom score | PR *v.s.* PD | 11/20 (55) | 14/19 (74) | 25/39 (64) |
| | PR *v.s.* NR | 11/28 (55) | 19/25 (76) | 30/52 (58) |
| | TTF>6 *v.s.* TTF<6 | 14/28 (50) | 18/25 (72) | 32/52 (62) |
| >10% ↑ in ALPBI+ >10% ↑ in BGP | PR *v.s.* PD | 15/22 (68) | 16/17 (94) | 31/38 (82) |
| | PR *v.s.* NR | 15/24 (63) | 28/29 (97) | 43/52 (83) |
| | TTF>6 *v.s.* TTF<6 | 16/24 (67) | 23/29 (79) | 39/52 (75) |
| >10% ↑ in ALPBI+ >10% ↓ in urinary calcium excretion | PR *v.s.* PD | 15/23 (65) | 12/13 (92) | 27/36 (75) |
| | PR *v.s.* NR | 15/25 (60) | 24/25 (96) | 39/50 (78) |
| | TTF>6 *v.s.* TTF<6 | 16/25 (64) | 19/15 (76) | 35/50 (70) |
| >10% ↑ in BGP+ >10% ↓ in urinary calcium excretion | PR *v.s.* PD | 14/18 (78) | 17/19 (89) | 31/37 (84) |
| | PR *v.s.* NR | 14/23 (61) | 27/29 (93) | 41/52 (79) |
| | TTF>6 *v.s.* TTF<6 | 15/23 (65) | 22/29 (76) | 37/52 (71) |
| >10% ↑ in ALPBI+ >10% ↑ in BGP+ >10% ↓ in urinary calcium excretion | PR *v.s.* PD | 14/18 (78) | 17/19 (89) | 32/36 (89) |
| | PR *v.s.* NR | 14/18 (78) | 30/32 (94) | 44/50 (88) |
| | TTF>6 *v.s.* TTF<6 | 14/18 (78) | 24/32 (75) | 38/50 (76) |
| >10% ↑ in ALPBI+ >10% ↑ in BGP+ >10% ↓ in urinary+ calcium excretion + >10% ↓ in symptom score | PR *v.s.* PD | 9/11 (82) | 18/25 (72) | 27/36 (75) |
| | PR *v.s.* NR | 9/11 (82) | 32/39 (82) | 41/50 (82) |
| | TTF>6 *v.s.* TTF<6 | 9/11 (82) | 26/39 (67) | 35/50 (70) |

PR = partial response, PD = progressive disease, NR = no response (no change + progressive disease); TTF>6 = time to progression>6 months, TTF<6 = time to progression <6 months; [a]See methods for definition of predictive values and diagnostic efficiency.

off of a 10% change from baseline (increase for ALP-BI and BGP, decrease for urinary calcium excretion and symptom score) was selected. From Table II it is clear that the PV− of single biochemical parameters is high but the PV+ and DE are relatively low. Combining parameters improved the discrimination and a combination of a >10% rise in ALP-BI and BGP wth a >10% fall in calcium excretion produced the highest DE. The DE with this combination was 89% for discriminating between response (PR) and progression (PD), 88% between PR and no response (PD plus no change NC) and 76% between TTF of >6 months and TTF of <6 months. The addition of symptom score did not improve the DE further.

Figure 5a and 5b show the discriminant effect of ALP-BI, BGP and urinary calcium excretion in Venn diagrams. The same cut-offs have been used. The intersection of the three sets in (5a) contains 14/16 responding patients but only 3/23 with progressive disease ($P<0.001$). In 5b all 53 patients are represented and divided into two groups according to time to progression. (TTF>6 months at the top and <6 months below). The intersection of the three sets contains only 4/31 patients progressing within 6 months compared with 14/22 patients relapsing after this time ($P<0.001$).

### Discussion

Metastatic bone destruction results from malignant cells in the bone marrow cavity secreting paracrine factors which stimulate osteoclasts to resorb bone (Mundy, 1987). In lytic metastases, the normal coupling between osteoblast and osteoclast function is disturbed and bone resorption predominates. Control of the tumour by systemic therapy reduces osteoclast activity and allows bone healing, mediated by osteoblasts, to occur. This study has shown that biochemical monitoring of bone cell function can predict eventual radiological response.

Response to therapy resulted in a flare in osteoblast activity and reduction in the rate of bone resorption, changes which were significantly different from those seen with progressive disease. A transient rise in ALP-BI and BGP, reaching a peak at 1–2 months occurred. As the response continued both parameters fell; the fall in BGP occurring more slowly reflecting continued remineralization. A transient rise in alkaline phosphatase during the first month of treatment has been noted before (Hortabagyi *et al.*, 1984) but this was not seen in all responders, possibly because isoenzyme measurements were not performed. In another study (Coombes *et al.*, 1983) changes in ALP were unhelpful but repeat measurements were not made until 2–4 months, a time when osteoblast activity is falling again.

The flare in osteoblast activity is seen also on serial bone scans performed during the first 6 months of treatment (Rossleigh *et al.*, 1984, Coleman *et al.*, 1986). This results in a paradoxical deterioration of the bone scan appearances in responding patients characterised by increased activity in baseline lesions and the appearance of new foci of increased tracer uptake. These findings are discussed in more detail elsewhere (Coleman *et al.*, 1988).

Control of metastatic disease led to a reduction in urinary calcium excretion as the rate of bone resorption slowed. The use of the calcium excretion index (Peacock *et al.*, 1969) has been reported previously as a marker of response (Campbell *et al.*, 1983) and we confirm their results. No patient with

**a** **b**

**Figure 5** Biochemical discrimination of response shown in Venn diagrams. Set A are patients showing a >10% rise in ALP-BI, Set B a >10% rise in BGP and Set C a >10% fall in urinary calcium excretion (all after 1 month of treatment). In (a) partial responders ($n=16$) are compared with progressive disease ($n=23$) and in (b) TTF>6 months ($n=22$) compared with TTF<6 months ($n=31$). (A=rise in ALP-BI, B=rise in osteocalcin, C=fall in calcium excretion).

responding disease or static disease for more than 6 months had a rise in calcium excretion at 1 month. Changes in the serum calcium within the normal range were unable to predict response but the development of hypercalcaemia during therapy invariably indicated progressive disease.

Increased bone resorption leads to an increase in hydroxyproline excretion but in this study serial measurements correlated poorly with response; a rise was seen in 73% of non-responders but levels fell in only 50% of responders. Similar results have been noticed previously (Coombes *et al.*, 1983) and attributed to either methodological problems with the assay or contribution from dietary and extra-skeletal sources.

Acid phosphatase is produced by several cell types but isoenzyme measurements enable indirect assessment of osteoclast activity. The tartrate-resistant phosphatase (TRP) isoenzyme is mainly derived from osteoclasts (Efstrdiatis & Moss, 1985) and metastatic bone disease causes levels to rise (Yarrison *et al.*, 1976). Although levels did not often fall as a result of effective therapy a rise was more common in progressive disease ($P<0.02$). Further study of this marker of bone metabolism in monitoring therapy may be worthwhile.

The relief of symptoms is the principal aim of palliative therapy and objective response correlated well with symptomatic response. Measurement of symptoms is both difficult and poorly standardised. The pain questionnaire used here was completed by the patient and provided a semi-quantitative assessment of pain, mobility and analgesic use. From these data a symptom score was easily computed. Patients responding to therapy showed a steady improvement in symptoms while patients progressing had at best only a slight and transient relief of symptoms, usually attributable to local radiotherapy. Worsening of symptoms indicated progressive disease.

The ability of a specified change (>10%) from baseline in the values of ALP-BI, BGP and urinary calcium excretion to predict response after 1 month of treatment was determined by calculating the predictive values (PV+ and PV−) and diagnostic efficiency (DE). The PV− of a single test was high, but the more clinically useful PV+ and thus the DE were relatively low. However, the combination of >10% rise in ALP-BI and BGP and >10% fall in urinary calcium excretion increased PV+, with little effect on PV− and hence, increased the DE. The DE of this combination was 89% for discriminating response (PR) from progression (PD). The use of this predictive model was extended to patients with radiologically stable disease by adding this group to those with PD as non-responders (DE 88%) or divided, according to the time to treatment failure (TTF), between responding and progressing patients (DE 76%).

In conclusion, biochemical monitoring appears to be a good alternative to plain radiography early on in treatment as it provides an indication of response long before radiological changes can be expected. We suggest using a >10% rise in ALP-BI and BGP with a 10% fall in calcium excretion after 1 month of treatment as an index for predicting response. However, our conclusions are based on a retrospective analysis of the data and it is recognised that independent prospective confirmation of our results is necessary.

We wish to thank Ms. Sue Hoare for hydroxyproline measurements, Mr George Chik for routine urine biochemistry and the nursing staff of the Hedley Atkins Unit for their help with sample collection.

### References

CAMPBELL, F.C., BLAMEY, R.W., WOOLFSON, A.M.J. *et al.* (1983). Calcium excretion $Ca_E$ in metastatic breast cancer. *Br. J. Surg.*, **70**, 202.

COLEMAN, R.E. & RUBENS, R.D. (1985). Bone metastases and breast cancer. *Cancer Treat. Rev.*, **12**, 251.

COLEMAN, R.E. & RUBENS, R.D. (1987). The clinical course of bone metastases from breast cancer. *Br. J. Cancer*, **55**, 61.

COLEMAN, R.E., RUBENS, R.D. & FOGELMAN, I. (1988). The bone scan flare following systemic therapy for bone metastases. *J. Nucl. Med.*, (in press).

COOMBES, R.C., DADY, P., PARSONS, C. *et al.* (1983). Assessment of response of bone metastases to systemic treatment in patients with breast cancer. *Cancer*, **52**, 610.

EFSTRDIATIS, T. & MOSS D.W. (1985). Tartrate resistant acid phosphatase of human lung: Apparent identity with osteoclastic acid phosphatase. *Enzyme*, **33**, 34.

GALEN, R.S. & GAMBINO, S.R. (1975). How to determine the predictive value and efficiency of a test when reading a scientific paper. In *Beyond Normality: The predictive value and efficiency of medical diagnosis*, Galen, R.S. & Gambino, S.R. (eds) p. 30. John Wiley & Sons: New York.

GRANT, C.S., HOARE, S.A., MILLIS, R.R., HAYWARD, J.L. & WANG, D.Y. (1984). Urinary hydroxyproline and prognosis in human breast cancer. *Br. J. Surg.*, **71**, 105.

HAYWARD, J.L., CARBONE, P.P., HEUSON, J.C., KUMOAKA, S., SGALOFF, A. & RUBENS, R.D. (1977). Assessment of response to therapy in advanced breast cancer. *Eur. J. Cancer*, **13**, 89.

HORTABAGYI, G.N., LIBSHITZ, H.I. & SEABOLD, J.E. (1984). Osseus metastases of breast cancer. Clinical, biochemical, radiographic and scintigraphic evaluation of response to therapy. *Cancer*, **55**, 577.

MOSS, D.W. & WHITBY, L.G. (1975). A simplified heat-inactivation method for investigating alkaline phosphatase isoenzymes in serum. *Clin. Chim. Acta.*, **61**, 63.

MUNDY, G.R. (1987). The hypercalcaemia of malignancy. *Kidney International*, **31**, 142.

PALAZZO, S., LIGUORI, V. & MOLINARI, B. (1986). Is the carcinoembryonic antigen test a valid predictor of response to medical therapy in disseminated breast cancer. *Tumori*, **72**, 515.

PEACOCK, M., ROBERTSON, W.D. & NORDIN, B.E.C. (1969). Relation between serum and urinary calcium with particular reference to parathyroid activities. *Lancet* **i**, 384.

PRICE, P.A., PARTHEMORE, J.G. & DEFTOS, L.J. (1980). New biochemical marker for bone metabolism. Measurement by radioimmunoassay of bone GLA protein in the plasma of normal subjects and patients with bone disease. *J. Clin. Invest.*, **66**, 878.

RANSOHOFF, D.F. & FEINSTEIN, A.R. (1978). Problems of spectrum and bias in evaluating the efficacy of diagnostic tests. *N. Engl. J. Med.*, **299**, 926.

ROSSLEIGH, M.A., LOVEGROVE, F.T.A., REYNOLDS, P.M. *et al.* (1984). The assessment of response to therapy of bone metastases in breast cancer. *Aust. NZ. J. Med.*, **14**, 19.

YARRISON, G., MERTENS, B.F. & MATHIES, J.C. (1976). New diagnostic use of bone marrow acid and alkaline phosphatases. *Am. J. Clin Pathol.*, **66**, 667.